# Effects of dose-dependent chronic caffeine consumption in a rat burn wound model: Histopathological and immunohistochemical evaluation 

**DOI:** 10.22038/IJBMS.2024.76513.16557

**Published:** 2024

**Authors:** Filiz Yilmaz, Selma Aydemi̇r, Bayram Yilmaz, Orkun Ilgen, Sefa Kurt, Başak Baykara

**Affiliations:** 1 Hitit University, Training and Research Hospital, IVF Center, Corum, Turkey; 2 Dokuz Eylul University, Faculty of Medicine, Department of Histology and Embryology, Izmir, Turkey; 3 Hitit University, Training and Research Hospital, Pathology Department, Corum, Turkey; 4 Dokuz Eylul University, Faculty of Medicine, Department of Obstetrics and Gynecology, Izmir, Turkey

**Keywords:** Caffeine, Integrin, MMP-9, VEGF, Wound healing

## Abstract

**Objective(s):**

Using histopathological and immunohistochemical methods, we aimed to examine the dose-dependent effects of chronic caffeine consumption on the recovery of burn wounds in an *in vivo* rat model.

**Materials and Methods:**

Forty-five rats were randomly assigned to a high-dose group (20 mg/kg per day for eight weeks; n=15), a low-dose group (10 mg/kg per day for eight weeks; n=15), or a control group (n=15). The burn model was created in rats. The groups were separated into three subgroups (n=5) based on the day after injury (7^th^, 14^th^, or 21^st^ day). The wound area, wound closure percentage, and histopathological and immunohistochemical reactivity were evaluated.

**Results:**

Successful wound healing was noted in rats treated with low doses of caffeine, similar to the control group. Pathology revealed low re-epithelization, low inflammation, and high granulation in the high-dose group. In addition, there was a significant difference between the control and high-dose groups regarding the immunohistochemical reactivity of αVβ3 integrin, vascular endothelial growth factor (VEGF), and matrix metalloproteinase 9 (MMP-9) (*P<*0.05).

**Conclusion:**

We demonstrated that chronic caffeine consumption in rats adversely affects the recovery process of wounds in a dose-dependent manner. This effect may occur through delayed wound healing via the molecules MMP-9, αVβ3 integrin, and VEGF. Treatment that modulates these molecules can lead to enhanced and quicker recovery of damaged skin in coffee lovers.

## Introduction

Post-injury events consist of three continuous phases and definite boundaries that cannot separate beginnings and ends. These stages can be classified as inflammatory, proliferative, and remodeling stages. In the inflammatory stage, the vascular response to stimulation starts with wound formation, accompanied by and a cellular response. The proliferation phase is characterized by extracellular matrix deposition and fibroblast migration to the wound site. Finally, the remodeling phase is the stage that shapes the structural durability and functional adequacy of the formed repair tissue (1).

Various cellular activities are required for wound healing and are stimulated or inhibited by tissue integrity mediators such as HB-EGF, VEGF, MMP-9, and integrins. Heparin-binding epidermal growth factor (HB-EGF) affects many stages of the wound recovery process, such as re-epithelization, granulation, and formation of damaged tissues and remodeling, wound contraction, and angiogenesis. Wound healing studies have shown marked impairment of wound closure in HB-EGF-deficient mice (2). Angiogenesis is stimulated by VEGF secreted from epidermal keratinocytes during wound healing. In this way, blood flow is provided, new blood vessels are generated in the injured area, and the recovery phase is accelerated. VEGF, which stimulates monocyte chemotaxis, increases the levels of growth factors derived from endothelial cells and is effective at facilitating coagulation. In addition, it enables granulocyte-macrophage precursor cells to form colonies (3). As a result of VEGF receptor activation, some intracellular signal transduction proteins are phosphorylated, thereby ensuring the proliferation, migration, differentiation, and inhibition of endothelial cell apoptosis. VEGF is the main factor regulating the vascularization and permeability of the wound area and is involved in granulation production during the proliferation phase (4). Many studies have investigated the therapeutic effects of VEGF on wound healing (5). These studies showed that granulation deposition increases due to the overproduction of VEGF.

In wound healing, destruction events, such as the remodeling phase, are as important as construction. Collagen breakdown at this stage is regulated mainly by matrix metalloproteinases (MMPs)(6). MMP activity is regulated by the tissue inhibitor metalloproteinase (TIMP). TIMP provides tight control of proteolytic activity within the scar by creating a natural counterbalance against the MMP. Any disruption of this balance results in excessive or insufficient matrix formation or an open wound. Integrins and their ligands play a primary role in the formalization, modulation, and remodeling of the ECM related to wound healing (7). Integrins bind to ECM proteins such as fibronectin and collagen. Various integrin subunits play regulatory roles in fibroblasts at wound sites. An increase in α5 integrin enhances fibroblast migration and may improve wound healing. A study has also shown that α5 integrin plays a crucial role in fibroblast proliferation in the wound area (8). During the wound healing process, αvβ3 modulates the structure and stability of the fibrin network, which is necessary for neoangiogenesis and plays a role in collagen attachment to the endothelium, endothelial cell survival, pericyte retention in blood vessels, and fibroblast proliferation. Studies have shown that the integrin αvβ3 molecule is vital for tissue remodeling. For example, it plays a role in activating MMP and various growth factors and in cell proliferation, migration, and differentiation (9).

Coffee, tea, chocolate, coke, and some carbonated beverages are regularly consumed daily and contain caffeine. It is known that caffeine consumption negatively affects the wound-healing process (10). However, the molecular mechanism of this process is unknown. In an *in vitro* study, caffeine dose-dependently restricted keratinocyte proliferation, and scratch wound healing experiments on keratinocyte monolayers showed that cell migration lags in a dose-dependent manner. Surprisingly, the differentiation and adhesion processes were unaffected in a dose-independent manner in the monolayer cultures. In an *in vitro* wound healing model study, topical caffeine application inhibited epithelization and these findings validated other *in vitro* data. As a result, caffeine inhibits keratinocyte proliferation and migration, wound healing, and epithelization (10). In addition, previous studies showed caffeine to induce apoptosis and inhibit cell proliferation (11, 12).

*In vitro* studies have shown the negative effects of caffeine consumption on the burn wound healing process. However, there is no precise information about the molecular mechanism involved in this process. In addition, these effects have not been tested *in vivo*. Therefore, our study aimed to examine the impacts of chronic dose-dependent caffeine consumption on the wound healing process in rats and evaluate the involvement of the HB-EGF, VEGF, integrin αvβ3, and MMP-9 molecules in different phases of wound healing using immunohistochemical methods.

## Materials and Methods

***Rats***

This scientific study was approved by the Dokuz Eylul University Local Ethical Committee of Animal Experiments (Protocol No. 35/2019). Therefore, the study was conducted in January 2020 at the Experimental Animal Laboratory, Dokuz Eylul University, Türkiye.

Our study employed 45 Wistar rats (6-8 months old, weighing 180-220 g). All rats were placed in cages at the appropriate temperature (22 ^°^C), humidity, and food and water availability and were subjected to a 12-hour light/dark cycle.

***Experimental protocol***

Our study was designed as a prospective experimental study. Forty-five animals were divided into three random groups: the control group (n=15), low-dose group (n=15), and high-dose group (n=15). Then, each group was divided into three subgroups, and specimens were taken from the rats on certain days (7^th^, 14^th^, and 21^st^) to assess the wound healing stages. Therefore, nine subgroups, including five rats, were generated (13).

Rats were given caffeine (Sigma‒Aldrich, No. C0750) dissolved in 1 ml of distilled water (10 mg/kg/day) as a low dose and 20 mg/kg/day as a high dose via oral gavage (14). The 20 mg/kg day caffeine exposure in rats equaled 1.5-2.2 cups of caffeine consumed by a human daily (each cup contained 100-150 mg). Caffeine was administered for eight weeks, representing the chronic caffeine consumption pattern of the rats (15). A wound model was created after 48 days of caffeine application in the caffeine groups. Thus, the first sample was taken from rats that consumed caffeine for at least eight weeks.

Rats were anesthetized with xylazine HCL (subcutaneous, 13 mg/kg) and ketamine HCL (subcutaneous, 90 mg/kg), and the wound model was created. First, the skin on the backs of the rats was shaved with a shaver without irritation, disinfected with batticon, and subsequently washed with normal saline. A metal device (diameter: 5x2.5 cm²) was used for the burn wound model. The plants were heated in 100 ^°^C boiling water for five minutes and the water was applied to the dorsal skin of the rats. Afterwards, the rats were weighed for 10 sec to induce a second-degree burn (16). Carprofen (subcutaneous, 4 mg/kg, once a day) was given for analgesia. Next, the wound area was washed with normal saline, covered with antibiotic cream daily in all groups, and left open to dry air.

After the burn model was created, the wound sites were photographed with a ruler to macroscopically evaluate wound recovery at 7, 14, and 21 days (17). The rats were sacrificed on different days after wound healing (7^th^, 14^th^, and 21^st^ days), after which the wound healing stages were evaluated (18,19).

***Burn wounds’ gross measurements and closure rates***

The burn wound area and closure rate were evaluated using photographs taken during the post-wound period on the 7^th^, 14^th,^ and 21^st^ days. First, the length (cm), width (cm), and area (cm^2^) of the wounds were measured with the ImageJ program (16). The mean wound closure (%) was subsequently calculated with the formula described by Oryan (20). Finally, the burn wound closure rate was calculated as the mean wound closure percentage (%).

***Excision of skin biopsies***

A total of 1 cm×1 cm of the wound site was removed at a total thickness of 3 mm from the middle part of the wound area. For histological examinations, the tissue samples were fixed in 10% neutral formalin solution, dehydrated in graded ethanol, cleared in xylol, embedded in paraffin, and then divided into 5 μm thick sections.

***Histological examination***

The sections were stained with hematoxylin and eosin (H&E) to evaluate the healing process of burn wounds and were compared on days 7, 14, and 21. Morphological and immunohistochemical analyses were simultaneously performed using a double-headed light microscope by three researchers to eliminate bias. An Olympus BX 51 digital camera (Tokyo, Japan) was used for taking images. Wound healing differs between rodents and people, mainly through contracture rather than epithelialization. Thus, the wound closure percentage is less critical than the other measures. In this consideration, further analyses were conducted on different parameters, such as new epithelial formation, inflammation, angiogenesis, ulceration, and collagen formation. These morphological parameters were used to evaluate the maturity of the wound repair process based on a previously reported system (21).

***Immunohistochemistry***

The wound healing ability of all the samples during the specified periods (7^th^, 14^th^, and 21^st^ days) was evaluated via immunohistochemistry. The sections were incubated at 4 ^°^C overnight with the following monoclonal antibodies: anti-VEGF (Cat. No: bs-1665R), anti-MMP-9 (Cat. No: bs-4593R), anti-αVβ3 integrin (Cat. No: bs-1310R), and anti-HB-EGF (Cat. No: bs-3576).

A hematoxylin solution was used to analyze nuclear staining, while a digital video camera (DP71; Olympus) was used to take images. The captured images were subsequently transferred to a computer and studied with a computer-assisted image analyzer system and a microscope (BX51; Olympus, Tokyo, Japan). A semiquantitative method was used to evaluate the immunohistochemical staining. The staining was divided into four main categories: strong (+++, 3), moderate (++, 2), weak (+, 1), or ambiguous (-, 0). Slides were examined by two histologists and one pathologist (22).

***Statistical analysis***

The Statistical Package for the Social Sciences (SPSS) 23 (SPSS, Inc., Chicago, IL, USA) was used for statistical analyses. A *P*-value<0.05 was considered to indicate statistical significance. The normality test was used for the data. Nonparametric statistical methods were chosen because they did not show a normal distribution. The values ​​are presented as mean±standard deviation (SD). One-way analysis of variance (ANOVA) was used to evaluate wound area and wound closure. The Kruskal–Wallis test was used for comparing immunohistochemical staining and morphological parameters. In addition, the Mann‒Whitney U test was performed to test the significance of pairwise differences using Bonferroni correction to adjust for multiple comparisons (16, 20).

**Figure 1:**
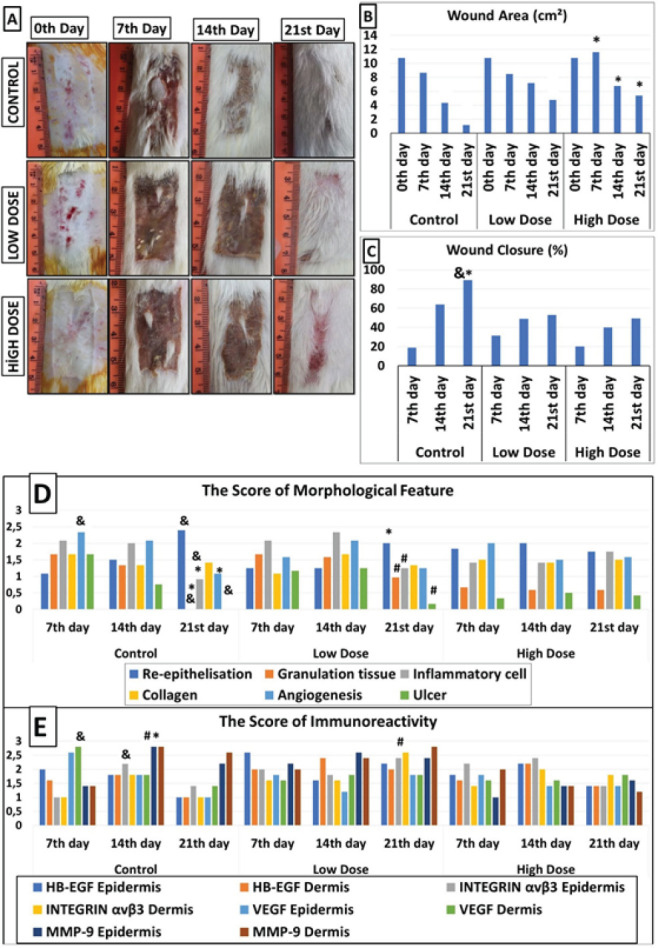
A. Effects of dose-dependent caffeine consumption on the burn wound healing process on days 0, 7, 14, and 21. B. Mean wound area (mm2) after burn injury in the control, low-dose, and high-dose groups of rats. C. Mean wound closure (%) after burn damage in the control, low-dose, and high-dose groups. D. Semiquantitative histomorphometric scores of the wounds (re-epithelized collagen, accumulated inflammatory cells, granulation tissue, angiogenesis, and ulcers) on post-wounding days 7, 14, and 21 in the animals (*P<0.05 compared with the control, low-dose and high-dose groups). (& *P<*0.05 compared with the control subgroup. % *P<*0.05 compared with the low-dose subgroup). E. Semiquantitative immunohistochemical staining of wound areas (HB-EGF, integrin, MMP-9, and VEGF) on post-wounding days 7, 14, and 21 in animals (*P<0.05 compared with the control, low-dose and high-dose groups). (& *P<*0.05 compared with the control subgroup. # *P<*0.05 compared with the low-dose subgroup). ANOVA was used to compare wound area and wound closure. The Kruskal–Wallis test was performed to evaluate the immunohistochemical staining and morphological parameters. Mann-Whitney U test was used for dual comparisons

HB-EGF=Heparin-binding epidermal growth factor, Integrin αVβ3=integrin alpha 5 beta 3, VEGF=vascular endothelial growth factor, MMP-9=matrix metalloproteinase-9

**Figure 2:**
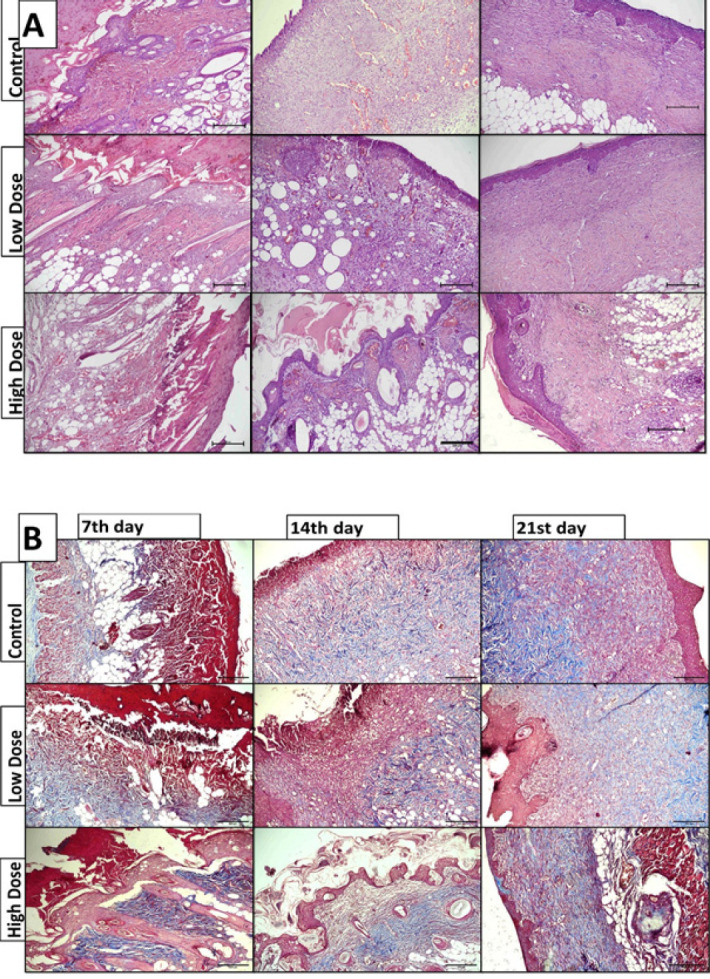
Wound histology of the control, low-dose, and high-dose group wounds at post-wounding days 7, 14, and 21. A. Hematoxylin and eosin staining, B. Masson trichrome staining (TRI); 10x magnification; scale bar: 200 µm). The morphological parameters (re-epithelization of collagen, accumulation of inflammatory cells, granulation tissue, angiogenesis, and ulceration) were used to evaluate the maturity of wound repair in H&E and TRI sections

**Figure 3:**
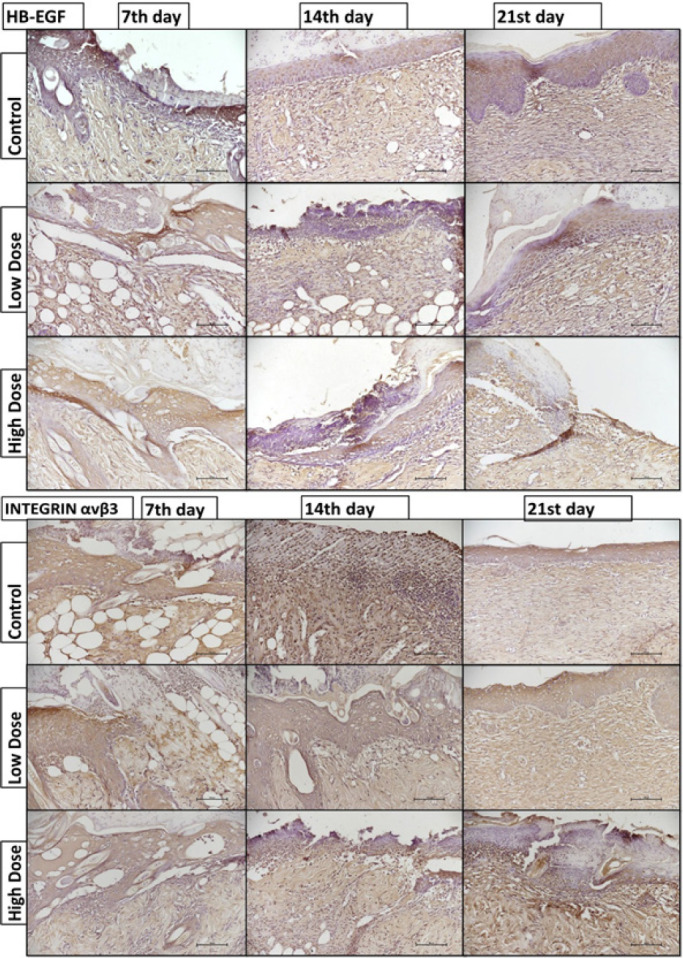
HB-EGF and integrin αVβ3 immunoreactivity results (x20 magnification, scale bar: 100 µm). HB-EGF Heparin-binding epidermal growth factor. The staining was divided into four main categories: strong (+++, 3), moderate (++, 2), weak (+, 1), or ambiguous (−, 0)

HB-EGF = Heparin-binding epidermal growth factor. integrin αVβ3= Integrin alpha 5 beta 3

**Figure 4:**
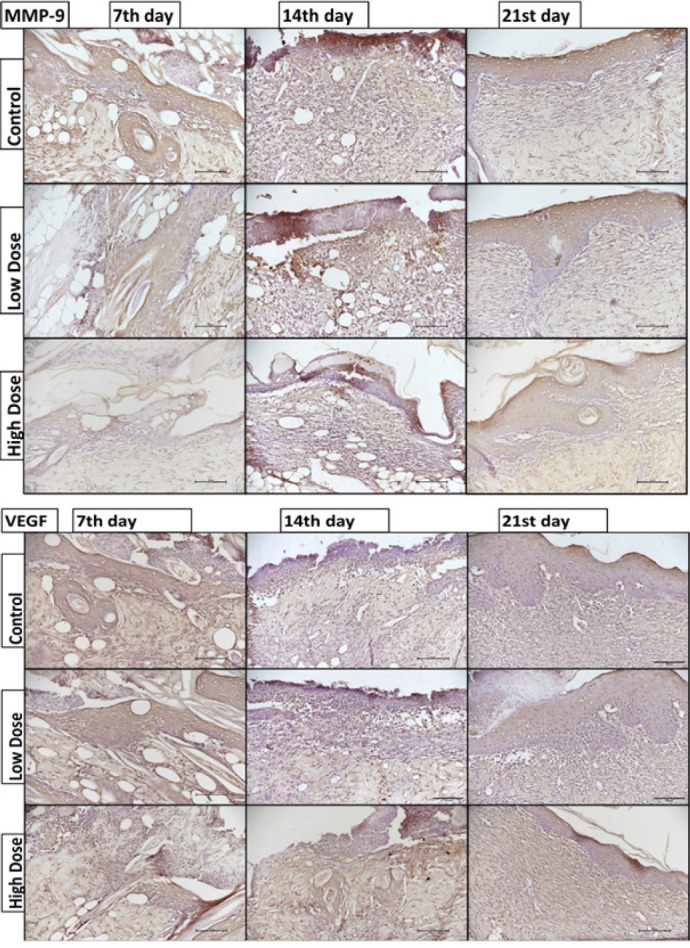
VEGF (A) and MMP-9 (B) immunoreactivity results (x20 magnification, scale bar: 100 µm). VEGF vascular endothelial growth factor, MMP-9 matrix metalloproteinase-9. The staining was divided into four main categories: strong (+++, 3), moderate (++, 2), weak (+, 1), or ambiguous (−, 0)

VEGF = vascular endothelial growth factor, MMP-9= matrix metalloproteinase-9

## Results

***Macroscopic findings***

Figure 1A shows the impacts of different dosages of caffeine consumption on burn wound recovery at 7, 14, and 21 days post-wounding. On days 7, 14, and 21, there was no evidence of redness, hyperemia, or inflammation in the control group wounds, while redness and inflammation were still observed in the high-dose groups (Figure 1A). The low-dose group exhibited mild inflammation and redness at 7, 14, and 21 days post wounding.

The same wound area was measured at 10.7±2.9 cm² on day 0 of wound healing in all rats in the control and caffeine groups. The mean wound areas were 8.6±5.3 cm² (7^th^ day), 4.1±1.3 cm² (14^th^ day), and 1.1±0.3 cm² (21^st^ day) in the control group; 8.4±0.7 cm² (7^th^ day), 7.1±1.3 cm² (14^th^ day), and 4.7±0.7 cm² (21^st^ day) in the low-dose group; and 11.5±0.4 cm² (7^th^ day), 6.7±1.3 cm² (14^th^ day), and 5.3±0.9 cm² (21^st^ day) in the high-dose group. The wound areas were significantly larger in the high-dose group than in the control and low-dose groups on days 7, 14, and 21 (*P<*0.05) (Figure 1B). No obvious differences were observed between the control and low-dose groups.

As shown in Figure 1C, the mean wound closure (%) was significantly greater in the control group than in the low- and high-dose groups (89.5±4.1, 52.9±16.7, and 49.3±24.2, respectively, on day 21; *P*<0.05). In addition, the control group’s wound closure rate was significantly greater on the 21^st^ day than on the 14^th^ and 7^th^ days (18.8±15.4, 63.7±17.4, and 89.5±4.1, 7^th^, 14^th^, and 21^st^ days, respectively; *P*<0.05). No significant difference was detected in the wound healing rate between the low-dose and high-dose groups on the 7^th^, 14^th^, or 21^st^ days post-wounding (*P>*0.05).

On the 21^st^ day of wound healing, wound healing was not completed in the high-dose caffeine group compared with the other groups (Figure 1A-C). Empirically, high doses of caffeine inhibited wound healing in a dose-dependent manner.

***Histopathological findings***

H&E and Masson’s trichrome staining were used for histopathological examination of the skin wounds on days 7, 14, and 21, as shown in Figure 2. The semiquantitative scores of the morphological features were calculated (Figure 1D).

***Re-epithelization***

On the 21^st^ day, the group with the highest rate of re-epithelialization was the control group. In the control group, the re-epithelization ratio on day 21 was greater than that on the 7^th^ and 14^th^ days (*P*<0.05). Similarly, in the rats that were taken up at a low level, the re-epithelization ratio increased as the number of wound healing days progressed (*P*<0.05) (Figure 2, 1D). Wound healing was evaluated as successful in the control and low-dose groups. However, there was no significant difference in re-epithelization between the high-dose subgroups (7^th^, 14^th^, and 21^st^ days). These findings indicated a delay in wound healing in the high-dose group.

***Collagen deposition***

Collagen deposition did not significantly differ between the groups. These findings demonstrated that chronic caffeine consumption did not affect collagen deposition in a dose-dependent manner (Figure 2, 1D).

***Inflammatory cells and granulation tissue***

On the 21^st^ day, the lowest inflammation and granulation tissue percentage among the groups was observed in the control group (*P*<0.05). In the control rats, unlike those on the re-epithelization treatment, the inflammatory cell and granulation tissue percentages were lower on the 21^st^ day than on the 7^th^ and 14^th^ days (*P*<0.05). Similarly, in the low-dose group, the inflammatory cell and granulation tissue percentages decreased as the number of wound healing days progressed (*P*<0.05)(Figure 2, 1D). However, no significant difference in inflammatory cell or granulation tissue percentages was detected between the high-dose subgroups.

***Ulcer***

The ulcer concentration decreased as the number of wound healing days progressed in the control group and similarly in the low-dose group (*P*<0.05 for both)(Figure 2, 1D). A reduction in the ulcer rate demonstrated successful wound healing. However, there was no significant difference in the ulcer rate between the high-dose subgroups. These findings indicated that wound healing was incomplete in the high-dose group.

***Angiogenesis***

Angiogenesis was high on the 7^th^ and 14^th ^days in the control group but decreased significantly on day 21 (*P*<0.05). However, there was no significant difference between the low- and high-dose subgroups (Figure 2, 1D). Our findings suggested that angiogenesis, which is high in the early stage of wound healing under physiological conditions, decreases in the late phase (when wound healing is complete). The absence of increased angiogenesis in the caffeine groups, particularly in the early stage, showed that chronic caffeine consumption negatively affects angiogenesis.

***Immunohistochemical analysis***

HB-EGF, integrin αVβ3, VEGF, and MMP-9 immunoreactivity were calculated on the 7^th^, 14^th^, and 21^st^ days for all groups (epidermis and dermis). The immunohistochemical score data are shown in Figure 1E.

HB-EGF immunoreactivity did not change significantly between the groups. Integrin immunoreactivity was highest on the 14^th^ day in the control group and highest on the 21^st^ day in the low-dose group (*P*<0.05). However, there was no significant difference in integrin immunoreactivity in the high-dose group on the 7^th^, 14^th^, or 21^st^ day (Figure 3).

VEGF immunoreactivity was the highest on day 7 in the control group, while it was lower on the other days (14^th^ and 21^st^) (*P*<0.05). However, no significant difference was detected in VEGF immunoreactivity between the low- or high-dose groups on 7^th^, 14^th^, or 21^st^ days (Figure 4).

MMP-9 immunoreactivity in the control group increased significantly on the 14^th^ day compared to that on the other days (7^th^ and 21^st^ days) (*P*<0.05). Conversely, compared with that in the other groups (control and low-dose groups), the MMP-9 expression in the high-dose group was significantly lower (*P*<0.05) on days 7, 14, and 21 (Figure 4).

## Discussion

The recovery process of burn wounds is complicated by the proliferative, inflammatory, and remodeling phases (1). The burn area depth and size and external factors are the most important parameters influencing mortality and morbidity after thermal injury. The burn depth and size cannot change, but external factors can be modified or affected. Therefore, knowing which external factors are beneficial or harmful and what mechanism they act through is essential. Caffeine is also an important external factor that is consumed daily and affects many physiological or pathological processes.

A previous study using *in vitro *and *ex vivo* models suggested that caffeine may inhibit wound healing and epithelization (10). In another study, carotid artery injury models were created, aged, and chronically induced, and different caffeine concentrations were used. Caffeine attenuates vascular damage-induced neointimal hyperplasia by suppressing smooth muscle cell proliferation in rats (14). Our study examined the dose-dependent effects of chronic caffeine consumption on burn wound recovery in an *in vivo* rat model. Our macroscopic results supported the findings of *in vitro* studies of caffeine. In the high-dose group, the appearance of the wound bed was consistent with incomplete wound healing, resulting in a delayed healing process compared to that in the control and low-dose groups. The wound bed was smaller in the control and low-dose groups than in the control group and wound closure was almost complete after three weeks.

Our study evaluated re-epithelialization, angiogenesis, granulation tissue, and histopathologic scores of inflammatory cells and wound closure (contraction). These selected parameters are indicators of wound healing. Rahman *et al*. created a burn wound model and examined the effect of amniotic gel on the burn healing process with similar histopathological scoring (23). In another similar study, an increase in inflammatory cells was observed in the early stage of wound healing in the control group. In contrast, an increase in granulation, angiogenesis, and collagen was observed, while a decrease in inflammatory cells was found in the advanced stages (24). Our histological results showed an increase in angiogenesis, inflammatory cells, and granulation in the early stages, while there was a decrease in these parameters in the advanced stages in the control group. The wound remodeling revealed an immature healing process characterized by fragmental re-epithelization, persistent fibrinous exudation, and few inflammatory cells, even after three weeks in the high-dose group. In contrast, complete re-epithelialization and the absence of granulation tissue were observed in the control group, with the low-dose group indicating findings similar to those of the control group.

Studies have shown that caffeine has antifibrogenic, anti-inflammatory, and antioxidant effects (24-26). For example, in a hepatotoxicity model, the effects of caffeine (37.5 mg/kg, eight weeks) were examined, concluding that the histological structure and functions of the liver improved. Caffeine has antifibrogenic, anti-inflammatory, and antioxidant effects (25). Wadhwa *et al*. showed that caffeine reduces inflammation by suppressing proinflammatory cytokines (27). An increase in inflammatory cells, which should be observed in the early phase of the wound recovery process, was not observed in the high-dose caffeine group in our study. This finding was considered a failure in wound healing. The inflammation-suppressing properties of high-dose chronic caffeine consumption may also explain this difference.

A study showed that caffeine *in vitro* prevents cell proliferation, cell migration, epithelization, and wound closure (10). In our *in vivo* study, we confirmed/supported these findings. Furthermore, we demonstrated that chronic caffeine consumption in rats negatively affects wound healing in a dose-dependent manner *in vivo*. Finally, immunohistochemical methods were used to evaluate the molecules that play a role in these negative effects.

Growth factors play roles in proliferation, angiogenesis, and granulation tissue formation and are fundamental for wound recovery (28). The present study analyzed HB-EGF and VEGF (growth factors). VEGF plays many roles in angiogenesis, which is vital in skin regeneration (29). In their studies, researchers showed that caffeine consumption affects VEGF expression (30). Similarly, in our study, the highest VEGF immunohistochemical staining was observed in the burned skin on day 7 in the control group, and the VEGF immunoreactivity decreased on days 14 and 21. However, neither an increase nor a decrease in VEGF intensity was observed in the high-dose group. We interpreted our findings that VEGF expression decreased due to the completion of wound healing on the 21^st^ day in the control group, but did not decrease due to delayed wound healing in the high-dose group. Delayed healing by other mechanisms may prevent a reduction in VEGF levels.

HB-EGF controls keratinocyte migration during wound healing, contributes to re-epithelization, and accelerates wound healing (31). Okonkwo *et al*. showed that HB-EGF mRNA levels were high on the 7^th^ and 14^th^ days and low on the 21^st^ day in the nonburn wound model they created (32). In our study, we created a burn wound model and evaluated the immunohistochemical reactivity of HB-EGF. Similar immunoreactivity levels were observed in the control group on days 7, 14, and 21 during the wound healing period. Similarly, the same immunoreactivity intensity was recorded in the subgroups of the low- and high-dose groups. As a result, we showed that HB-EGF plays a role in all phases of burn wound recovery. Additionally, caffeine consumption does not affect the HB-EGF level in a dose-dependent manner.

Integrin αvβ3 is expressed during wound healing and mediates fibroblast adhesion and migration via different ECM proteins. Angiogenesis within the healing wound is necessary for tissue regeneration and is determined by integrin-mediated adhesion to the ECM (33). A study reported high integrin αvβ3 immunofluorescence reactivity on the 7^th^ and 14^th^ days and low integrin αvβ3 immunofluorescence on the 21^st^ day in a wound model created in rabbits (34). In our study, integrin reactivity peaked on the 14^th^ day in the control group and decreased on the 21^st^ day as wound healing was completed. However, integrin immunoreactivity remained unchanged in the high-dose group. We inferred that the reason for this lack of integrin immunoreactivity was incomplete wound recovery.

MMP-9 has an intricate role in wound recovery, such as epithelial regeneration, remodeling, ECM remodeling, angiogenesis, and inflammation. Previous studies have noted that MMP-9 improves inflamed skin in response to damage and the need for tissue remodeling (35). Nagy *et al*. determined the importance of MMP-9 for wound healing (36). Our previous study revealed that a decrease in MMP-9 and integrin expression plays a role in the negative effect of caffeine on embryo implantation in a dose-dependent manner (37). A study investigated the effects of caffeine on invasion and the anticancer impact on human glioblastomas *in vitro*. Caffeine decreases the invasion of glioblastoma cells In addition, it increases the mRNA and protein expression of tissue inhibitors of metalloproteinase-1 (TIMP-1), whereas MMP-2 is down-regulated (38). In another study, caffeine was shown to reduce the invasion of human leukemia U937 cells and decrease the protein and mRNA expression levels of MMP-2 and MMP-9 (39). In our study, MMP-9 immunoreactivity was low in the high-dose group. Insufficient MMP-9 prevented the re-establishment of the dermal-epidermal junction in the high-dose group, limiting epithelial migration and wound closure.

Our study revealed that MMP-9, VEGF, and integrin αvβ3 expression was disrupted or abnormal in chronic high-dose caffeine groups. Caffeine could adversely affect wound healing through these three molecules.

## Conclusion

The present study has several limitations, including small sample sizes and a small number of doses. It is challenging to talk about dose dependency with only two doses of caffeine studied. We aimed to include more than two groups, but the number of animals sacrificed would increase significantly. Therefore, we limited animal mortality by checking with fewer groups.

A better understanding of burn wound healing pathophysiology can reduce burn complications and enable the discovery of alternative treatment methods. In addition, negative external factors such as caffeine consumption affecting this process should be excluded. If negative external factors cannot be modulated, their action mechanisms must be considered and additional treatments should be planned for these mechanisms.

These findings may guide the elucidation of the pathogenesis of the dose-dependent adverse effects of chronic caffeine consumption on wound healing. For people who consume excessive caffeine, it is advisable to consider adding an agent to treatment that can support burn wound healing and reduce the adverse effects of caffeine. Additional studies of alternative treatment methods for burn wound healing are needed and clinical studies should also be conducted.
